# Association between circadian syndrome and chronic diarrhea: a cross-sectional study of NHANES 2005–2010 data

**DOI:** 10.3389/fphys.2024.1301450

**Published:** 2024-04-29

**Authors:** Liang Ding, Jinnan Duan, Tao Yang, Chaoqiong Jin, Shanmei Lv, Ahuo Ma, Yuehua Qin

**Affiliations:** ^1^ Department of Gastroenterology, Shaoxing People’s Hospital, Shaoxing, China; ^2^ Department of Infectious Diseases, Shaoxing People’s Hospital, Shaoxing, China; ^3^ Department of Clinical Laboratory, Shaoxing People’s Hospital, Shaoxing, China

**Keywords:** circadian syndrome, chronic diarrhea, bowel health, sleep, National Health and Nutrition Examination Survey

## Abstract

**Background:**

Circadian rhythms are reported to influence physiological processes in the gastrointestinal system, but associations between circadian syndrome (Circs) and chronic diarrhea (CD) remain unclear. Here, we explored such relationships to provide new insights into CD management.

**Methods:**

We conducted a cross-sectional retrospective analysis using the National Health and Nutrition Examination Survey (NHANES) data between 2005 and 2010. Univariate and multivariable logistic regression analyses were performed on weighted data to explore associations between Circs and CD.

**Results:**

Results were presented using forest plots, odds ratios (ORs), and 95% confidence intervals (CIs). Data with *p*-values < 0.05 were considered statistically significant. In total, 5,661 US participants, of which 412 had CD (weighted percentage = 6.20%), were enrolled. In univariate logistic regression analyses, participants with Circs had a significantly higher risk of CD (OR = 1.51, 95% CI: 1.15–1.99). After adjusting for covariates, model 2 (OR = 1.40, 95% CI: 1.03–1.90) and model 3 (OR = 1.42, 95% CI: 1.01–2.00) data were consistent with model 1 data. Additionally, the number of Circs components was positively associated with CD in all three models. Subgroup analyses revealed an association between CD and Circs in participants who had high blood pressure (OR = 2.46, 95% CI: 1.48–4.11, *p* < 0.001).

**Conclusion:**

In this cross-sectional study, we found that Circs is positively associated with the risk of CD in US adults, especially in those with high blood pressure. This association may provide new management strategies for CD.

## 1 Introduction

Chronic diarrhea (CD) is the second most common gastrointestinal symptom that affects approximately 5% of adults worldwide (Schiller et al., 2017). Prevalence rates vary from countries, partly through population differences but also through definition. In two studies from the United States and Japan, which defined chronic diarrhea as Bristol Stool Form Scale (BSFS) types 6–7, the prevalence of chronic diarrhea in the general population was 6.6% and 3.0%, respectively (Singh et al., 2018; Matsumoto et al., 2022). In two other studies from China and Norway, which defined chronic diarrhea by Rome II standard, the prevalence of chronic diarrhea was 3.26% and 8.8%, respectively (Fosnes et al., 2011; Zhao et al., 2012). The characterization of CD is refractory and recurrent, which can significantly reduce the quality of life by causing fatigue, depression, and increasing hospitalizations and health costs (Zhang et al., 2022). Current treatment efficacy is not optimal and may be associated with complex pathogenic mechanisms, especially in functional diarrhea where potential impact factors include dietary choices, intestinal flora, and chronic inflammation (Black et al., 2020).

Circadian rhythms refer to regular cyclical patterns established by intracellular clock genes and physiological responses to environmental signals (Hu et al., 2022). Rhythms are represented by self-sustained oscillations in many biological systems that persist in the absence of external 24-h cues (Meyer et al., 2022). A canonical property of circadian rhythms is entrainment, where circadian systems use environmental cues (known as zeitgebers and include light, temperature, and behavioral processes such as locomotor activity and feeding) to adjust for deviations in period and control phases in internal rhythms (Mohawk et al., 2012; Masuda et al., 2021). In contemporary society, circadian misalignment has emerged as a significant detrimental factor, where our lifestyles increasingly incorporate circadian risk factors, such as electronic devices, air conditioners, shift work, international travel, and staying up late. These disruptions to natural circadian rhythms can have negative consequences on our metabolic health, leading to an increased risk of inflammatory, cardiovascular, neurological, and malignant diseases (Sobolewska-Włodarczyk et al., 2016). For bowel health, recent evidence has confirmed that circadian rhythms can influence the gut microbiota and microbial composition in terms of driving inflammation and hormonal responses (Teixeira et al., 2020; Lotti et al., 2023). Population-based studies have also highlighted close relationships between circadian rhythms and bowel health, e.g., shift work appears to partly increase the risk of functional gastrointestinal disease (Koh et al., 2014), peptic ulcer disease (Knutsson and Bøggild, 2010), and reflux esophagitis (Nam et al., 2023).

Circadian syndrome (Circs) may be manifested as seven different traits, namely, abnormal blood pressure, fasting glucose, waist circumference, triglycerides, high-density lipoprotein (HDL) cholesterol (HDL-C), short sleep sessions, and depression, and is based on the evidence that circadian disruption may underpin metabolic risk factors and disorders (Zimmet et al., 2019; Shi et al., 2021). However, despite our growing understanding of circadian-related health issues, no previous research has yet explored the relationships between Circs and CD.

To address this, we used the National Health and Nutrition Examination Survey (NHANES) database to investigate potential links between Circs and CD. By examining data from a large and diverse population sample, we investigated relationships to provide new insights for CD management.

## 2 Methods

### 2.1 Data source

The NHANES database is a cross-sectional, multistage, nationally representative survey that provides considerable information about dietary behavior, medical examinations, and the health status of the general US population. The NHANES protocol was approved by the National Center for Health Statistics Ethics Review Board, with written informed consent obtained from all participants. All NHANES data were de-identified.

### 2.2 The study cohort

**FIGURE 1:**
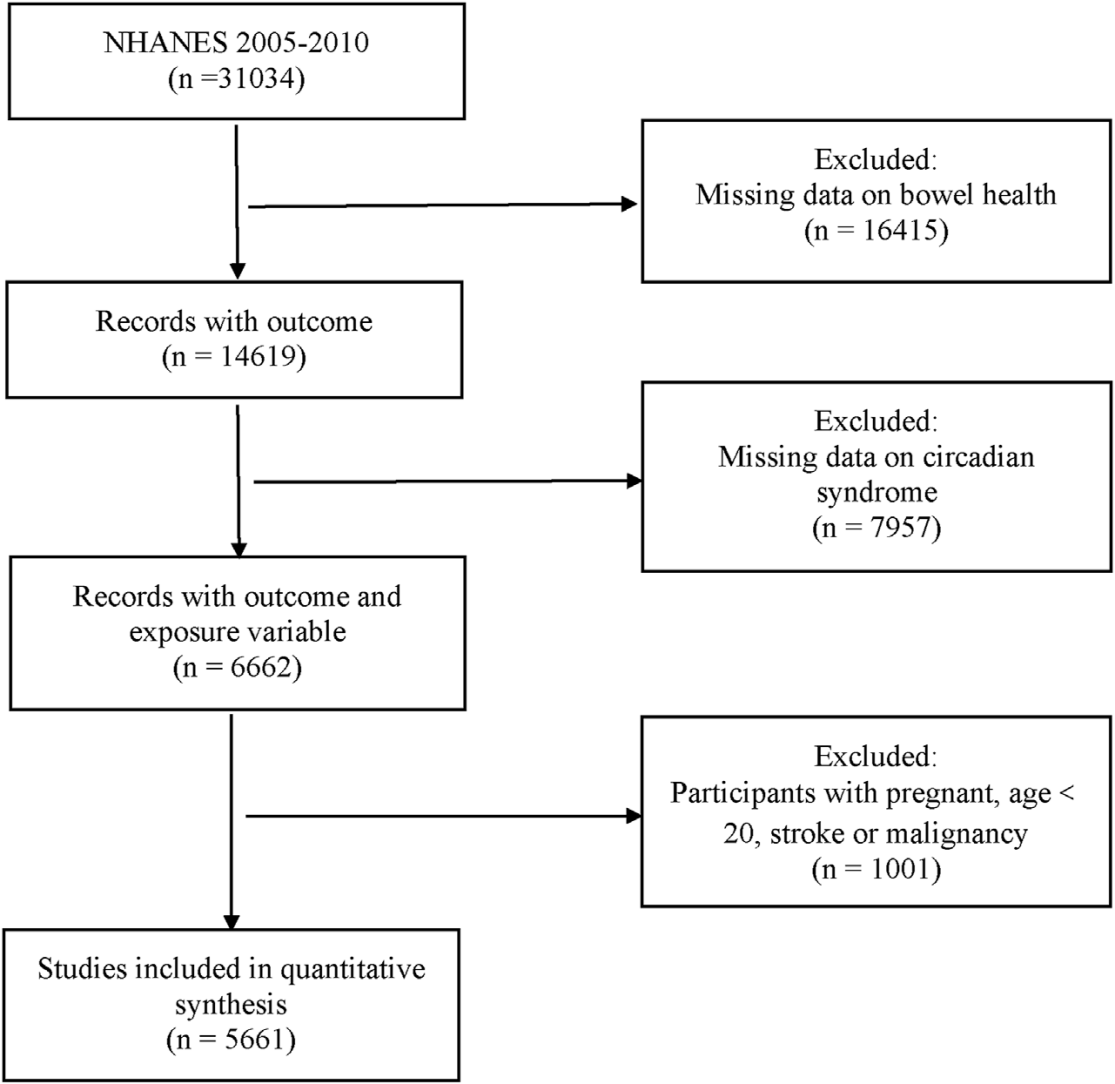
Flow diagram of the process for the selection.

Participants from three NHANES cycles (2005–2010) answered bowel health questionnaires, with Circs information gathered for this study. Any missing data relating to these items were excluded. Participants aged <20 years old were also excluded because the target cohort for bowel health questionnaires was only adults aged ≥20 years. Additionally, we excluded individuals who were pregnant and had a previous stroke or malignancy history (Figure 1).

### 2.3 Study variables

#### 2.3.1 CD (dependent variable)

Bowel health information was recorded in a dedicated bowel health questionnaire, which provided data on stool consistency using the Bristol Stool Form Scale (BSFS). As a validated and widely recognized measure, the BSFS provided a color-coded chart containing seven stool types; participants looked at cards and indicated a number that corresponded to their usual or most common stool type. CD was defined as BSFS types 6–7 and non-diarrhea as types 1–5 (Sommers et al., 2019).

#### 2.3.2 Circs (exposure variable)

Circs was defined as ≥ 4 components from the following diagnostic criteria (Shi et al., 2021): 1) waist circumference ≥88 cm in women or ≥ 102 cm in men; 2) triglycerides ≥150 mg/dL or the use of lipid-lowering medication; 3) HDL-C levels < 40 mg/dL in men or < 50 mg/dL in women or the use of lipid-lowering medication; 4) blood pressure, systolic ≥ 130 or diastolic ≥ 85 mmHg or the use of antihypertensive drugs; 5) fasting glucose of ≥ 100 mg/dL or the use of antidiabetic medication; 6) sleep duration of ≤ 6 h/day; and 7) depression symptoms where a patient health status questionnaire-9 indicated a score of ≥ 10.

### 2.4 Covariates

The following covariates were included: age, gender, race, education level, marital status, poverty income ratio, vigorous activity, smoking status, alcohol-intake status, and dietary and comorbidity factors. Race categories were divided into four groups: Mexican American, non-Hispanic White, non-Hispanic Black, and other race (including multiracial). Education levels were categorized into three groups: < high school level, high school and college level, or above. Poverty income ratios were grouped as < 1.3, 1.3–3.49, and ≥3.5 according to the US Census Bureau. Vigorous physical activity was defined as “vigorously intense activity that increased breathing or heart rates for at least 10 continuous minutes.” Smoking status was categorized based on the number of cigarettes smoked in a lifetime: <100 or ≥100 cigarettes. Alcohol consumption was classified as < 12 or ≥12 drinks/year. Dietary fiber, carbohydrates, fat, protein, sugar, and calorie intake were obtained from the first day of the 24-h dietary recall. Comorbidity was assessed using self-reported diagnoses from medical condition questionnaires or specific disease questionnaires. Prescription medications during a 1-month period were recorded in the prescription medication section (DSQ).

### 2.5 Statistical analyses

The NHANES uses sample weights to process/sample large numbers of subgroups to reflect the true relative proportion of the US population. The use of correct sample weights for the NHANES analyses depends on the variables. A robust approach is used where a variable collected for the smallest number of respondents refers to the least common denominator.

To account for our complex sampling design, all estimates were weighted after taking the primary sampling unit, pseudo-strata, and sampling weights. For categorical variables, data were presented as raw counts and weighted proportions [n (%)], and significance was calculated using weighted chi-squared tests. For continuous variables, data were presented as weighted medians (25% and 75%), and significance was calculated using weighted Wilcoxon rank-sum tests.

Three logistic regression models were performed to explore correlations between CD and Circs: model 1 involved univariate logistic regression analyses where only CD and Circs were considered; model 2 was adjusted for age, gender, race, education, marital status, poverty income ratio, and comorbidity; and given our knowledge about CD, model 3 was further adjusted for vigorous activity, smoking status, alcohol consumption, and dietary factors. Simultaneously, subgroup analyses based on differences in diagnostic components were performed to elaborate correlations. The results were presented in forest plots using odds ratios (ORs) and 95% confidence intervals (CIs).

Data collation and statistical analyses were conducted using R 4.3.1 software. A *p*-value <0.05 was considered statistically significant.

## 3 Results

### 3.1 Baseline characteristics

In total, 5,661 participants, including 412 with CD (weighted percentage = 6.20%), were enrolled. Baseline characteristics are shown (Table 1). Compared to the non-diarrhea group, participants with CD were older, had lower educational levels, more carbohydrate consumption, and had higher arthritis prevalence (*p* < 0.05). The remaining characteristics were well balanced between groups, including sex, race, smoking, and alcohol status.

**TABLE 1:** Baseline characteristics: a comparison by chronic diarrhea.

	Overall (*n* = 5,661)	Non-diarrhea (*n* = 5,249)	Chronic diarrhea (*n* = 412)	*p*-value
**Age (y)**	44.00 (32.00, 55.00)	44.00 (32.00,55.00)	46.00 (36.00, 57.74)	**0.043**
**Female**, **n (%)**	2,751 (49.09%)	2,519 (48.80%)	232 (53.44%)	0.171
**Race, n (%)**				0.120
Mexican American	1,109 (8.64%)	1,002 (8.42%)	107 (11.94%)	
Non-Hispanic White	2,649 (69.64%)	2,491 (69.93%)	158 (65.31%)	
Non-Hispanic Black	1,105 (11.06%)	1,023 (11.03%)	82 (11.47%)	
Other race	798 (10.66%)	733 (10.62%)	65 (11.28%)	
**Educational, n (%)**				**0.019**
Less than high school	1,609 (17.61%)	1,441 (17.13%)	168 (24.98%)	
High school	1,345 (24.29%)	1,260 (24.39%)	85 (22.78%)	
College or above	2,707 (58.10%)	2,548 (58.48%)	159 (52.24%)	
**Married, n (%)**	3,483 (65.35%)	3,228 (65.39%)	255 (64.67%)	0.789
**PIR**				0.083
≤1.3	1,550 (18.45%)	1,412 (18.20%)	138 (22.24%)	
1.3–3.49	2,034 (37.12%)	1,894 (36.86%)	140 (40.99%)	
≥3.5	1,658 (44.43%)	1,564 (44.94%)	94 (36.77%)	
**Vigorous activity, n (%)**	1,392 (27.66%)	1,296 (27.67%)	96 (27.45%)	0.942
**Smoking**	2,650 (46.36%)	2,445 (46.05%)	205 (51.04%)	0.102
**Alcohol**	4,117 (77.38%)	3,832 (77.43%)	285 (76.72%)	0.802
**Dietary factors (g)**				
Fiber	14.40 (9.70, 20.40)	14.40 (9.70, 20.30)	14.70 (9.80, 21.53)	0.401
Carbohydrates	241.92 (176.52, 327.52)	241.44 (176.06, 326.17)	263.76 (187.13, 347.47)	**0.038**
Fat	77.78 (51.68, 108.10)	77.79 (51.77, 107.71)	77.38 (47.71, 112.45)	0.804
Protein	79.46 (56.66, 107.71)	79.25 (56.66, 107.62)	81.62 (56.81, 108.68)	0.748
Sugar	102.19 (62.03, 154.32)	101.40 (61.82, 153.59)	112.75 (65.69, 166.45)	0.068
Calorie (kcal)	2,058.87 (1,526.00, 2,750.05)	2,048.31 (1,519.86, 2,748.71)	2,160.39 (1,611.61, 2,884.50)	0.129
**Comorbidity, n (%)**				
Asthma	754 (14.19%)	687 (13.98%)	67 (17.46%)	0.131
Heart disease	188 (2.53%)	170 (2.47%)	18 (3.47%)	0.263
Anemia	213 (3.29%)	197 (3.22%)	16 (4.23%)	0.438
Thyroid problems	480 (9.07%)	443 (8.99%)	37 (10.24%)	0.597
Arthritis	1,464 (23.87%)	1,330 (23.51%)	134 (29.30%)	**0.047**

Data are shown as n (%) or median (interquartile range), of which frequency counts were unweighted. *p*-value was calculated using the weighted Wilcoxon rank-sum test for continuous variables and weighted chi-square test for categorical variables.

y: years; PIR: poverty income ratio; g: gram; kcal: kilocalorie.

*p*-value < 0.05 are in bold.

### 3.2 Circs characteristics

In total, 1,758 participants were identified with Circs, of which 1,582 had non-diarrhea symptoms. The weighted Circs percentage was significantly different between CD and non-diarrhea groups (35.07% vs. 26.35%, *p* = 0.004). Compared to the non-diarrhea group, participants with CD had a higher prevalence of diagnostic components, including blood pressure, glucose, triglycerides, and depression; weighted percentages were 35.83% vs. 28.09%, 55.50% vs. 45.43%, 42.32% vs. 35.18%, and 11.73% vs. 5.81%, respectively, for both groups. Critically, these differences were statistically significant (*p* < 0.05) (Table 2).

**TABLE 2:** Characteristics of circadian syndrome components: a comparison by chronic diarrhea.

Positive variable	Overall (*n* = 5,661)	Non-diarrhea (*n* = 5,249)	Chronic diarrhea (*n* = 412)	*p*-value
BP	1,842 (28.57%)	1,665 (28.09%)	177 (35.83%)	**0.015**
Glu	2,908 (46.05%)	2,652 (45.43%)	256 (55.50%)	**0.007**
TG	2,192 (35.62%)	1,999 (35.18%)	193 (42.32%)	**0.011**
HDL-C	2,201 (36.47%)	2,007 (36.08%)	194 (42.38%)	**0.070**
SS	2,239 (36.87%)	2,078 (36.90%)	161 (36.32%)	0.871
DP	442 (6.17%)	379 (5.81%)	63 (11.73%)	**0.003**
WC	3,072 (51.80%)	2,809 (51.45%)	263 (57.13%)	0.072
Circs	1,758 (26.89%)	1,582 (26.35%)	176 (35.07%)	**0.004**

Unweighted frequency counts and weighted percentages are shown.

Statistic difference was calculated using the weighted chi-square test.

BP: blood pressure; Glu: glucose; TG: triglycerides; HDL-C: high-density lipoprotein cholesterol; SS: short sleep; DP: depression; WC: waist circumference; Circs: circadian syndrome.

*p*-value < 0.05 are in bold.

### 3.3 Circs associations with CD

**FIGURE 2:**
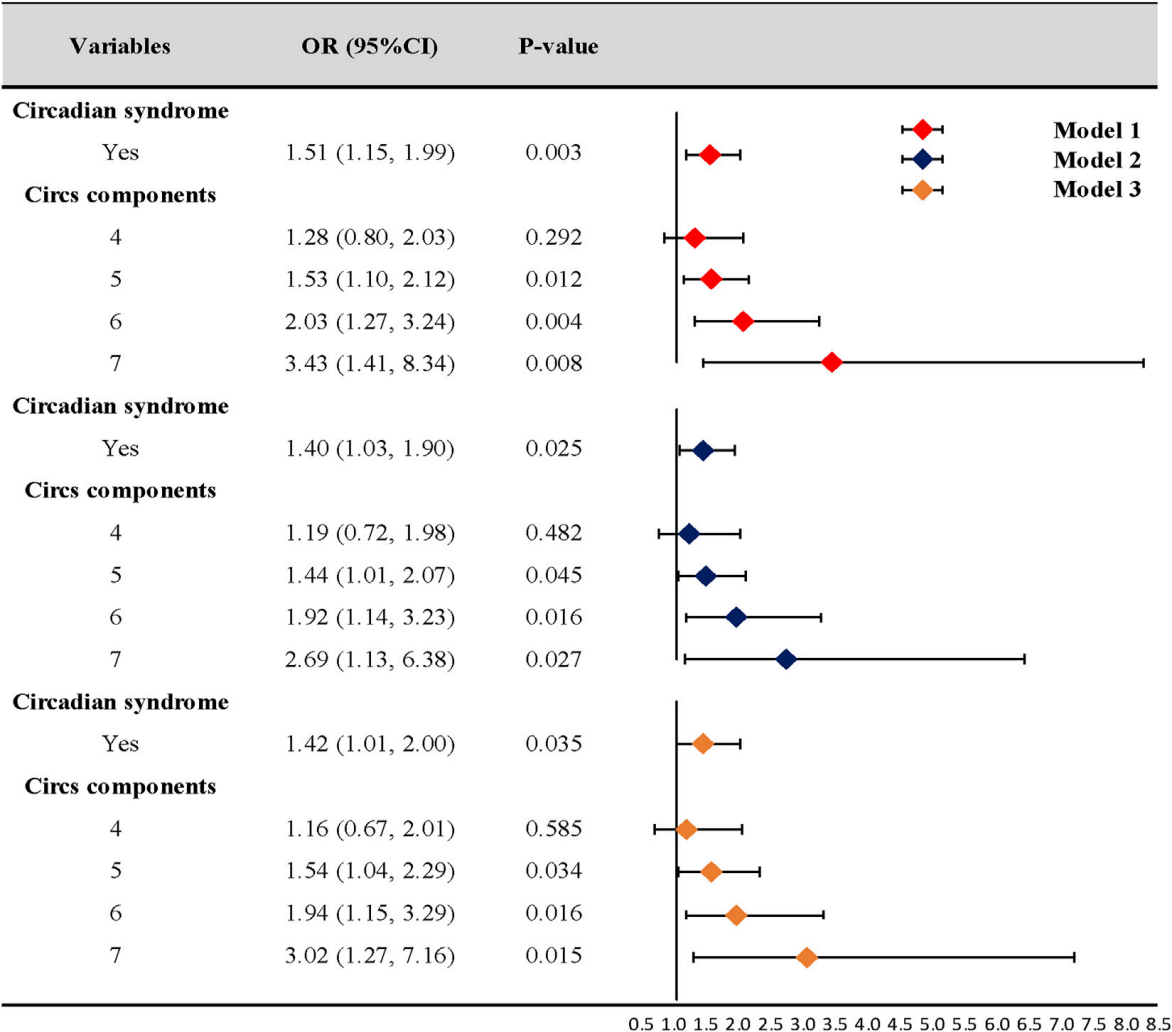
Association of circadian syndrome and chronic diarrhea. CI: confidence interval; OR: odds ratio. Model 1: univariate logistic regression analysis. Model 2: adjusted for age, gender, race, education, marital status, poverty income ratio, and comorbidity. Model 3: further adjusted for vigorous activity, smoking, alcohol consumption, and dietary factors based on model 2.

Our logistic regression analyses showed that the risk of CD was significantly higher in participants with Circs than in those without the condition (OR = 1.51, 95% CI: 1.15–1.99, *p* = 0.003). After adjusting for covariates, model 2 (OR = 1.40, 95% CI: 1.03–1.90, *p* = 0.025) and model 3 (OR = 1.42, 95% CI: 1.01–2.00, *p* = 0.035) data were consistent with model 1 data. Additionally, the number of Circs components was positively associated with CD in all analysis models (Figure 2).

Of note, very low numbers (*n* = 13) of respondents were taking medications that may have impacted bowel function (e.g., fiber supplements, stool-softening laxatives, and antidiarrheal medications). The risk of CD was still significantly higher in participants with Circs after excluding these cases (data shown in Supplementary Material).

### 3.4 Subgroup analysis

**FIGURE 3:**
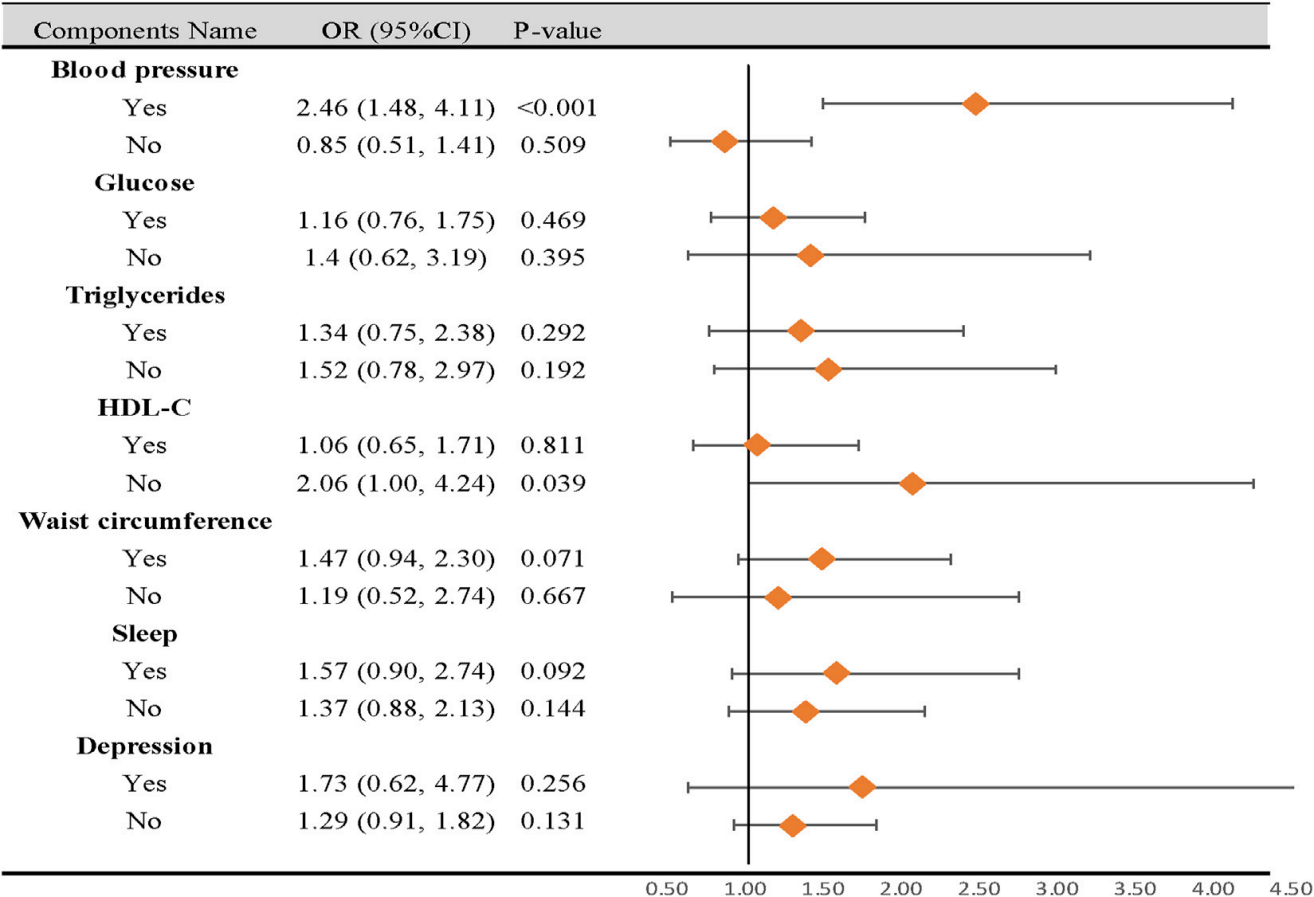
Subgroup analysis of the association between circadian syndrome and chronic diarrhea based on the components. CI: confidence interval; OR: odds ratio; Circs: circadian syndrome. Statistical analysis was performed in model 3.

In subgroup analyses, participants with Circs, with high-blood pressure characteristics, had a higher risk of CD than those with normal blood pressure (OR = 2.46, 95% CI: 1.48–4.11, *p* < 0.001). Additionally, participants with decreasing HDL-C characteristics were negatively correlated with a risk of CD (OR = 2.06, 95% CI: 1.00–4.24, *p* = 0.039). No differences in the remaining components were observed in subgroup analyses (Figure 3).

## 4 Discussion

In this cross-sectional study, we investigated relationships between Circs and CD using the NHANES database. Our results suggested that Circs was positively associated with CD prevalence, especially for participants with high-blood pressure characteristics.

Circadian rhythms have broad implications for human health, including metabolic, immunological, neurological, and gastrointestinal diseases, which remind us of the potential of circadian-based intervention and manipulation (Fishbein et al., 2021). In recent years, circadian misalignment has increasingly emerged as a potentially under-recognized public health problem due to modern lifestyle factors, such as exposure to artificial light at night, low indoor light levels during the day, extended hours of food consumption, controlled ambient temperatures, and shift work (Manfredini et al., 2018; Almoosawi et al., 2019). According to the Centers for Disease Control and Prevention, more than 33% of US individuals reported sleeping for less than 7 h in a day-and-night period (Liu et al., 2016), approximately 16% of adults worked on night shifts, and 28.7% worked alternative shifts (Alterman et al., 2013). However, the dilemma of diagnosing circadian disruption impedes the value to health reference in practical applications. Therefore, Circs consisting of biological indicators related to circadian rhythm disturbance may be used as effective measures to address these societal challenges (Zimmet et al., 2019). For example, Shi et al. (2021) and Shi et al. (2022) reported that Circs had a better predictive value than metabolic syndrome for cardiovascular disease in both Chinese and US adults. Additionally, studies also reported positive Circs associations with stroke (Wang et al., 2022), kidney stones (Xiao et al., 2023a), and testosterone deficiency (Xiao et al., 2023b).

In bowel research, different actions and mechanisms show circadian oscillation traits, e.g., clock genes and immunoreactivity were rhythmically expressed throughout the gastrointestinal myenteric plexus and in epithelial crypt cells (Hoogerwerf et al., 2007). Additionally, saliva production, gastric emptying, and colonic motor activity actions were weaker in the evening (Hoogerwerf, 2010). Moreover, relative gut microbiome abundance, microbiome proximity to the gut epithelium, and microbial metabolism all exhibited circadian rhythms (Zarrinpar et al., 2014). In our study, we observed an association between Circs and CD. This finding was generally consistent with previous studies. For example, Zhang et al. (2022) found that persons with chronic diarrheal symptoms had higher odds of sleep disorder by 26%. The state of rapid eye movement sleep, arousals, and waking was associated with increased colon motility that may induce the occurrence of diarrhea (Kim et al., 2018). However, positive associations were not identified between CD and short sleep, which is most directly related to disrupted circadian rhythms. Surprisingly, when we reviewed the literature, multiple studies reported no correlations between CD and sleep duration (Elsenbruch et al., 2002; Heitkemper et al., 2005; Zhang et al., 2022). Previous studies investigating the impact of sleep quality on human bowel health also reported contradictory results. Nojkov et al. (2010) observed that shift work was associated with mixed irritable bowel syndrome but not functional constipation and functional diarrhea in 399 nurses. Another study indicated an opposite result that abdominal symptoms, especially functional diarrhea, had a strong relationship with sleep disturbance incidences in 2,936 health-check subjects (Morito et al., 2014). Thus, it should be emphasized that short sleep was included as one of the Circs diagnostic components, and that a single component cannot adequately reflect circadian dysregulation. In our three-model analyses, OR increased with the number of diagnostic elements, consistent with a previous study (Wang et al., 2022), which meant that the number of matching characteristics increased the risk of CD. In future research, the precise quantification of representative elements in Circs may benefit its clinical management.

Previous NHANES studies reported a higher prevalence of CD in individuals with diabetes (11.2% vs. 6.0%) (Sommers et al., 2019), depression (15.53% vs. 6.05%) (Ballou et al., 2019a), sleeping for <4 h (8.4% vs. 7.6%) (Wang et al., 2022), and obesity (9.8% vs. 4.5%) (Ballou et al., 2019b). Given this evidence, it was important to consider the potential impact of single Circs components on CD risk, despite variations in definitions. Therefore, we performed subgroup analyses to explore the influence of single components. The results showed that participants with Circs, with high-blood pressure characteristics, had a higher risk of CD than those with normal blood pressure. Furthermore, no significant differences were identified for the remaining components in subgroup analyses, except for HDL-C levels. These findings concurred with our expectations: as stated, the OR increased with the summation of diagnostic components. Furthermore, these findings indicated that participants with Circs and CD may benefit by reducing their blood pressure. However, we were surprised to observe that participants with decreased HDL-C levels were negatively correlated with CD risks. This unexpected finding was possibly attributed to sample size limitations; therefore, further prospective studies are required to confirm this.

The main strength of our study was that we included a relatively large sample size, which improved the reliability of our results. To the best of our knowledge, few studies have explored the correlation between Circs and CD in a representative US adult sample. Circs is a new concept based on circadian rhythms, which reflects the potential adjustment of physiological activities and processes. Considering the high prevalence of CD and the recent emergence of Circs, our study provides clinical insights into CD management in affected populations. For example, we can attempt to recommend patients to improve diarrhea through regular schedules and rest habits. Additionally, our findings remind medical staff to care about the bowel health for patients with sleep problems.

However, our study had several limitations. First, due to the inherent limitations of a retrospective study, information bias and uncomplete data were inevitable. Furthermore, for variable definitions, we could not freely adjust NHANES data (e.g., smoking status). Likewise, it was difficult to clearly distinguish specific CD causes, which may have partly interfered with clinical interpretations. Furthermore, some clinical information was based on participant self-reporting. Although this was the only feasible approach for such a large epidemiological investigation, self-reporting may have introduced potential confounders and biases into our study. In future prospective studies, more detailed information should be provided about different diarrhea etiologies, especially functional diarrhea, to reinforce our results. Additionally, we failed to fully explain the direct relationship between circadian rhythms and CD, as described that Circs traits appear as surrogate markers to overcome the dilemma of diagnosing circadian disruption. However, recognizing the link between Circs and CD has an important implication for non-pharmacological prevention and therapeutic strategies of disease. Further research should examine the specific contribution of Circs to better understand the mechanisms underlying circadian rhythms and its association with CD.

## 5 Conclusion

In summary, in US adults, Circs was significantly associated with the risk of CD, particularly in participants with high blood pressure. These findings provide new insights into CD management, but more prospective studies are warranted to confirm these relationships.

## Data Availability

The original contributions presented in the study are included in the article/Supplementary Material; further inquiries can be directed to the corresponding author.
