# Supplementary material for: Association between circadian syndrome and chronic diarrhea: a cross-sectional study of NHANES 2005–2010 data
Source: Front Physiol. 2024 Apr 29;15:1301450. doi: 10.3389/fphys.2024.1301450 (PMC11089382; doi:10.3389/fphys.2024.1301450)
Supplement: Supplementary file 1 [file DataSheet1.PDF]

The association of circadian syndrome and chronic diarrhea

Variable	Model 1		Model 2		Model 3	
	**OR (95%CI)**	P	**OR (95%CI)**	P	**OR (95%CI)**	P
**Circadian syndrome**						
No	Ref		Ref		Ref	
Yes	1.51(1.15, 2.00)	0.003	1.40(1.03, 1.90)	0.023	1.43(1.01, 2.01)	0.033

CI: confidence interval; OR: odds ratio.

Model 1: univariate logistic regression analysis.

Model 2: adjusted for age, gender, race, education, marital status, poverty income ratio, and comorbidity.

Model3: further adjusted for vigorous activity, smoking, alcohol consumption and dietary factors based on model 2.
